# Lack of vitamin D signalling in mesenchymal progenitors causes fatty infiltration in muscle

**DOI:** 10.1002/jcsm.13448

**Published:** 2024-03-27

**Authors:** Tohru Hosoyama, Minako Kawai‐Takaishi, Hiroki Iida, Yoko Yamamoto, Yuko Nakamichi, Tsuyoshi Watanabe, Marie Takemura, Shigeaki Kato, Akiyoshi Uezumi, Yasumoto Matsui

**Affiliations:** ^1^ Department of Musculoskeletal Disease, Research Institute National Center for Geriatrics and Gerontology Obu Japan; ^2^ Department of Orthopaedic Surgery Nagoya University Graduate School of Medicine Nagoya Japan; ^3^ Department of Surgical Oncology The University of Tokyo Tokyo Japan; ^4^ Institute for Oral Science Matsumoto Dental University Nagano Japan; ^5^ Center for Frailty and Locomotive Syndrome National Center for Geriatrics and Gerontology Obu Japan; ^6^ Department of Orthopaedic Surgery National Center for Geriatrics and Gerontology Obu Japan; ^7^ Graduate School of Life Science and Engineering Iryo Sosei University Fukushima Japan; ^8^ Research Institute of Innovative Medicine Tokiwa Foundation Fukushima Japan; ^9^ Medical Institute of Bioregulation Kyushu University Fukuoka Japan

**Keywords:** Inter/intramuscular adipose tissue, Mesenchymal progenitor, Sarcopenia, Vitamin D, VDR

## Abstract

**Background:**

Recent studies have indicated the importance of muscle quality in addition to muscle quantity in sarcopenia pathophysiology. Intramuscular adipose tissue (IMAT), which originates from mesenchymal progenitors (MPs) in adult skeletal muscle, is a key factor affecting muscle quality in older adults, suggesting that controlling IMAT formation is a promising therapeutic strategy for sarcopenia. However, the molecular mechanism underlying IMAT formation in older adults has not been clarified. We recently found that the vitamin D receptor (VDR) is highly expressed in MPs in comparison to myotubes (*P* = 0.028, *N* = 3), indicating a potential role of vitamin D signalling in MPs. In this study, we aimed to clarify the role of vitamin D signalling in MP kinetics, with a focus on adipogenesis.

**Methods:**

MPs isolated from mouse skeletal muscles were subjected to adipogenic differentiation conditions with or without vitamin D (1α,25(OH)2D3, 100 nM) for 7 days, and adipogenicity was evaluated based on adipogenic marker expression. For *in vivo* analysis, tamoxifen‐inducible MP‐specific VDR‐deficient (*Vdr* ^MPcKO^) mice were newly developed to investigate whether lack of vitamin D signalling in MPs is involved in IMAT formation. To induce muscle atrophy, *Vdr* ^MPcKO^ male mice were subjected to tenotomy of the gastrocnemius muscle, and then muscle weight, myofibre cross‐sectional area, adipogenic marker expression, and fatty infiltration into the muscle were evaluated at 3 weeks after operation (*N* = 3–4). In addition, a vitamin D‐deficient diet was provided to wild‐type male mice (3 and 20 months of age, *N* = 5) for 3 months to investigate whether vitamin D deficiency causes IMAT formation.

**Results:**

Vitamin D treatment nearly completely inhibited adipogenesis of MPs through Runx1‐mediated transcriptional modifications of early adipogenic factors such as PPARγ (*P* = 0.0031) and C/EBPα (*P* = 0.0027), whereas VDR‐deficient MPs derived from *Vdr* ^MPcKO^ mice differentiated into adipocytes even in the presence of vitamin D (*P* = 0.0044, Oil‐Red O^+^ area). In consistency with *in‐vitro* findings, *Vdr* ^MPcKO^ mice and mice fed a vitamin D‐deficient diet exhibited fat deposition in atrophied (*P* = 0.0311) and aged (*P* = 0.0216) skeletal muscle, respectively.

**Conclusions:**

Vitamin D signalling is important to prevent fate decision of MPs towards the adipogenic lineage. As vitamin D levels decline with age, our data indicate that decreased vitamin D levels may be one of the causes of IMAT formation in older adults, and vitamin D signalling may be a novel therapeutic target for sarcopenia.

## Introduction

Sarcopenia is a multifactorial disease, and certain age‐related alterations in the body are associated with sarcopenia pathophysiology. Although low muscle quantity (muscle volume) affects sarcopenia onset and/or exacerbation, recent studies have indicated that age‐related changes in muscle quality are also important in the pathophysiology of this disease because impairment of muscle quality results in reduced muscle function.^1^ In fact, the European Working Group on Sarcopenia in Older People has suggested to add the term ‘muscle quality’ in the revised consensuses on the definition and diagnosis of sarcopenia.^2^ Although there is no universal consensus definition of muscle quality at present, myosteatosis and fibrosis have been raised as factors affecting muscle quality.^3^

Inter/intramuscular adipose tissue (IMAT) is defined as a fatty infiltration into the interstitial space between myofibres, and this distinctive pathology is frequently observed in older adults. IMAT has emerged as an important factor influencing muscle quality in older adults,^4^, ^5^ suggesting that controlling IMAT formation may be a promising therapeutic strategy for sarcopenia. However, the molecular mechanisms of IMAT formation in older adults have not been fully unravelled, and it remains to be clarified how fat is eroded in skeletal muscle and why IMAT is formed predominantly in older adults, but not in young people. Mesenchymal progenitors (MPs), also known as fibro/adipogenic progenitors, are located in the gaps between myofibres and are thought to be a source of IMAT because of their multipotent differentiation capacity, including adipogenesis.^6^, ^7^ In fact, adipogenesis or fibrogenesis of MPs occurs in some pathological conditions, including age‐related tissue impairment,^8^, ^9^ whereas in normal conditions, MPs play a role in the maintenance of muscle homeostasis as phalangeal‐like cells.^10^ Although the precise molecular regulatory mechanism has not been clarified, it has been demonstrated that Runx1 transcriptionally regulates the expression of early adipogenic factors such as PPARγ and C/EBPα in MPs.^11^

Vitamin D, a fat‐soluble vitamin, is an important nutrient for tissue homeostasis and critically contributes to calcium metabolism and the maintenance of musculoskeletal systems. One of vitamin D pathways is mediated by binding to the vitamin D receptor (VDR), and upon binding to the retinoid X receptor (RXR), the vitamin D/VDR/RXR complex directly regulates the transcription of target genes. In skeletal muscle, VDR is expressed in myogenic cell lineages, such as myoblasts and myofibres, and affects their dynamics and functions,^12^, ^13^ indicating that skeletal muscle is one of target tissues in the body. Vitamin D signalling contributes to muscle function through the regulation of muscle contraction,^13^ although further examinations are needed to fully elucidate the role of vitamin D in skeletal muscle. Vitamin D is endogenously produced in the skin upon exposure to sunlight and can be obtained from foods and dietary supplements. However, blood vitamin D level decrease with age and vitamin D deficiency is believed to be widespread worldwide,^14^ suggesting a relationship between reduced blood vitamin D levels and age‐related diseases such as osteoporosis and sarcopenia.^13^, ^15^ VDR is expressed in a wide variety of cell types in addition to myogenic cells,^16^ suggesting that vitamin D signalling via VDR is important for cellular physiology in various tissues/organs.

The present study built on our preliminary findings that VDR was expressed in mouse primary MPs and at higher levels than myogenic cells. As the role of vitamin D signalling in MP behaviours remained unknown, we aimed to clarify the functional role of vitamin D signalling in MPs, with a focus on adipogenesis, and whether defects in this signalling pathway in MPs are associated with IMAT formation.

## Methods

### Human subjects

All study participants provided written informed consent for all experimental procedures. The study was approved by an institutional ethics review board (no. 1557). Small pieces of the vastus medialis and vastus lateralis were obtained from 48 female patients with osteoarthritis (OA) who underwent total knee arthroplasty (*n* = 26) or total hip arthroplasty (*n* = 22) at the Department of Orthopaedic Surgery at the National Center for Geriatrics and Gerontology (Obu, Japan). Muscles were dissected in accordance with a previously reported institutional protocol.^17^ The average age of the patients who underwent quantitative IMAT analysis was 74.1 years (range, 52 to 86 years). Dissected muscles were frozen in liquid nitrogen‐chilled isopentane and stored at −80°C until use for the preparation of frozen muscle cross‐sections.

### Intramuscular adipose tissue quantification in frozen human muscle sections

Frozen thin (7 μm) muscle sections were subjected to immunofluorescence staining for perilipin (Cell Signalling Technology (CST), Danvers, MA, USA) and laminin (Thermo Fisher Scientific, Waltham, MA, USA), and haematoxylin & eosin staining following fixation with 4% paraformaldehyde. IMAT and muscle fibre areas in the sections were evaluated based on perilipin and laminin staining, respectively, and the IMAT area in each section was calculated using the ImageJ software (NIH, Bethesda, MD, USA).

### Animals

Young and aged male C57BL/6N mice were obtained from Clea Japan, Inc (Tokyo, Japan) and the NCGG aging farm (Obu, Japan),^18^ respectively. Vitamin D‐deficient (AIN‐93G‐based) and control (AIN‐93G containing 0.02% vitamin D) diets were obtained from Clea Japan and provided to both young (3 months of age) and aged (20 months of age) mice for 3 months (*N* = 5). Body weight/mouse and food consumption/cage were measured weekly. Mice were sacrificed after 3 months, and the hindlimb muscles were collected and weighed. The dissected muscles were frozen in liquid nitrogen‐chilled isopentane and stored at −80°C until use.

*Pdgfra* ^CreER^ (*Pdgfra* ^CE^) mice were obtained from the Jackson Laboratory (#018280) and *Vdr*‐floxed mice were obtained from the University of Tokyo.^13^, ^19^ These mice were crossed to generate *Vdr* ^MPcKO^ mice. To induce Cre‐mediated recombination for MP‐specific *Vdr* knockout, 9‐month‐old mice were injected intraperitoneally with 1 mg of tamoxifen per 10 g body weight for 5 days. Four weeks after tamoxifen injection, the mice were subjected to a tenotomy experiment.

All animal experiments were approved by the Institutional Animal Care and Use Committee of the National Center for Geriatrics and Gerontology (no. 4‐15, 4‐16, 5‐7, and 5‐8).

### Primary cell culture

Satellite cells and MPs were enriched from dissected mouse hindlimb muscles using a MACS magnetic separator (Miltenyi Biotech, Bergisch Gladbach, Germany). Satellite cells were separated using anti‐α7‐integrin antibody (MBL life sciences, Tokyo, Japan), and the α7‐integrin‐negative fraction was further separated using anti‐Sca‐1 antibody (Miltenyi Biotech). The isolated satellite cells and MPs were cultivated in Dulbecco's modified Eagle’ medium (DMEM) supplemented with 20% fetal bovine serum until differentiation induction. For myotube differentiation, SC‐derived myoblasts at 90% confluency were maintained in DMEM supplemented with 2% horse serum. For adipogenic differentiation, MPs were maintained in adipogenic differentiation medium (ADM) containing insulin, dexamethasone, and 3‐isobutyl‐1‐methylxanthine for 2 days and further cultivated in ADM containing insulin for 5–7 days (Takara Bio, Shiga, Japan).

Primary mouse MPs were subjected to immunofluorescence analysis for platelet‐derived growth factor alpha (PDGFRα; anti‐rabbit; CST) and VDR (anti‐rat; Thermo Fisher Scientific). Alexa Fluor 594‐labelled goat anti‐rabbit IgG or Alexa Fluor 488‐labelled goat anti‐rat IgG (CST) was used as the secondary antibody.

### Quantitative reverse transcription PCR (qPCR)

RNA was extracted from hindlimb muscles and primary cells using TRI Reagent (Molecular Research Center, Cincinnati, OH, USA) reverse transcribed using the PrimeScript II 1st strand cDNA synthesis kit (Takara Bio). qPCRs were run using a CFX96 Real‐Time PCR Detection System (Bio‐Rad, Hercules, CA, USA). The qPCR primers are listed in Table 1.

**Table 1:** Sequences of the primers used for qPCR

Gene	Forward (5′‐3′)	Reverse (5′‐3′)
*myoD*	AGCACTACAGTGGCGACTCA	GCTCCACTATGCTGGACAGG
*Pdgfra*	GACGAGTGTCCTTCGCCAAAGTG	CAAAATCCGACCAAGCACGAGG
*mKi67*	CCTTGGCTTAGGTTCACTGTCC	TGCAGAATCCAGATGATGGAGC
*Runx1*	GGCAACTAACTGCTGGAACT	CTCATCTTGCCGGGGCTCAG
*Pparg*	CCAGAGCATGGTGCCTTCGCT	CAGCAACCATTGGGTCAGCTC
*Cebpa*	AGCAACGAGTACCGGGTACG	TGTTTGGCTTTATCTCGGCTC
*aP2*	CCGCAGACGACAGGA	CTCATGCCCTTTCATAAACT
*Plin1*	CTGTGTGCAATGCCTATGAGA	CTGGAGGGTATTGAAGAGCCG
*Gapdh*	GTGAAGGTCGGTGTGAACG	ATTTGATGTTAGTGGGGTCTCG
*Vdr*	CCTCACTGGACATGATGGAACCG	GATGTAGGTCTGCAGCGTGTTGG
*Pref‐1*	TGGCTGTGTCAATGGAGTCTGC	CCACGCAAGTTCCATTGTTGGC
*Sox9*	CACACGTCAAGCGACCCATGAA	TCTTCTCGCTCTCGTTCAGCAG
*Klf2*	CACCTAAAGGCGCATCTGCGTA	GTGACCTGTGTGCTTTCGGTAG

### Western blot analysis

Protein was extracted from primary cells using radio immunoprecipitation assay buffer and subjected to sodium dodecyl sulfate polyacrylamide gel electrophoresis in Laemmli sample buffer. After transfer to a polyvinylidene difluoride membrane, target proteins were detected by western blotting using the standard method. The following primary antibodies were used for western blotting: anti‐PDGFRα (CST), anti‐VDR (9A7; Thermo Fisher Scientific), anti‐C/EBPα (CST), anti‐perilipin‐1 (CST), anti‐Runx1 (CST), and β‐actin (Proteintech, Rosemont, IL, USA).

### Cultivation of mouse mesenchymal progenitors with vitamin D or retinoic acid

Primary mouse MPs were cultivated in adipogenic differentiation medium with or without vitamin D (100 nM, calcitriol; Cayman Chemical, Ann Arbor, MI, USA) or ec23 (1 μM, Sigma‐Aldrich, St. Louis, MO, USA), which is a synthetic photostable retinoic acid, for 7 days. Then, the cells were fixed with 4% paraformaldehyde and subjected to Oil red O staining or immunofluorescence analysis using anti‐PPARγ antibody (Santa Cruz Biotechnologies, Dallas, TX, USA) as a primary antibody.

MPs derived from *Pdgfra* ^CE/wt^: *Vdr* ^flox/flox^ mice at 50% confluence were treated with hydroxy‐tamoxifen (100 mM, Sigma‐Aldrich) for 3 days to induce VDR deficiency. Then, the medium was changed to DMEM supplemented with 20% fetal bovine serum for additional few days. When the cells reached 100% confluence, the medium was replaced with adipogenesis induction medium containing vitamin D (100 nM).

### Tenotomy

Nine‐month‐old *Pdgfra* ^CE/wt^: *Vdr* ^flox/flox^ and *Vdr* ^MPcKO^ mice (*N* = 3) were anaesthetized with isoflurane and the distal tendons of the gastrocnemius and soleus muscles were transected.^20^ The incision was closed using a 4–0 nylon suture (Bear Medic Corporation, Ibaraki, Japan). Three weeks after tenotomy, atrophied gastrocnemius muscles were collected, and muscle weight and the cross‐sectional area were measured. Muscle weight was normalized to body weight.

### Statistical analysis

Data are presented as mean ± standard deviation. Means of two groups were compared using a two‐tailed Student's *t*‐test. Pearson's correlation coefficient was used to correlate % IMAT and age. The data were processed using GraphPad Prism version 7 or 9 (GraphPad Software, San Diego, CA, USA). Statistical significance was set at *P* < 0.05.

## Results

### Intramuscular adipose tissue in human muscle increases with aging

**Figure 1:**
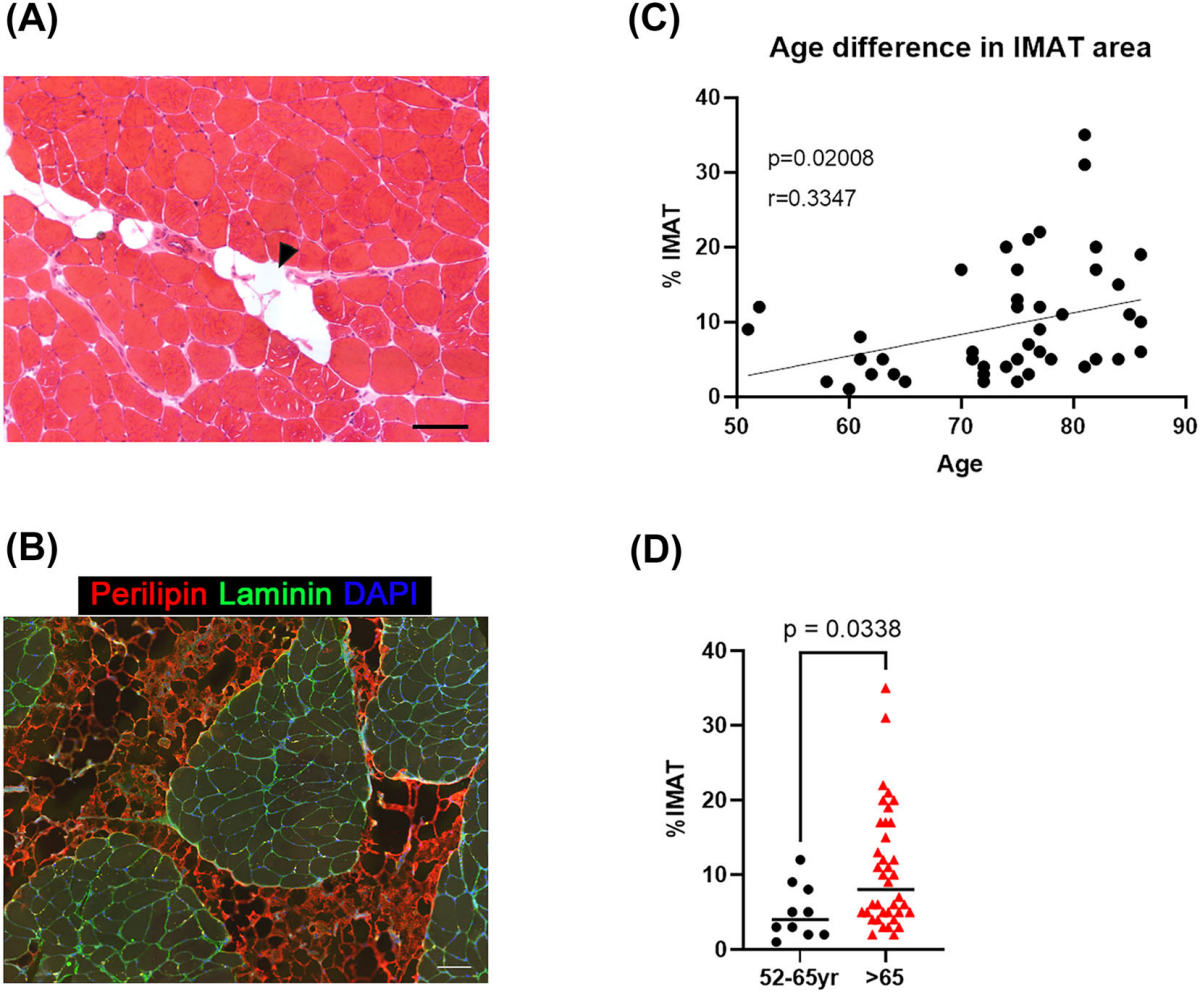
Intramuscular adipose tissue (IMAT) increases with age. (A) Representative haematoxylin & eosin staining image of vastus medialis muscle from a 70‐year‐old female. The arrowhead indicates IMAT. Scale bar = 100 μm. (B) Representative immunofluorescent image for perilipin and laminin from 81‐year‐old female. Scale bar = 100 μm. (C, D) The IMAT area was quantified based on the perilipin immunostaining‐positive area in a muscle cross section. IMAT infiltration was correlated with aging (*r* = 0.3347, *P* = 0.02008), and older adults (>65 years old) exhibited higher fat infiltration in the skeletal muscle (*P* < 0.05). As the World Health Organization and our country's laws define the older adults as 65 years of age or older, 65 years of age was used as the cut‐off value in this study.

We conducted histological analysis for IMAT to investigate whether aging was correlated with IMAT. Muscle cross sections from patients (52–86 years old, Figure 1A) were immunostained with anti‐perilipin antibody to visualize adipose tissue in the skeletal muscle, and the ratio of the perilipin^+^ IMAT area to the total area of each section was calculated for each patient (Figure 1B). There was a significant correlation between % IMAT and patient age (*P* = 0.02008, Figure 1C), and IMAT infiltration in the muscle was significantly higher in patients aged >65 years than the younger patients (Figure 1D). These results were consistent with those of a previous study using a non‐invasive imaging system (transverse ultrasonography),^21^ corroborating that aging is correlated with IMAT formation.

### Vitamin D receptor is highly expressed in mouse mesenchymal progenitors

**Figure 2:**
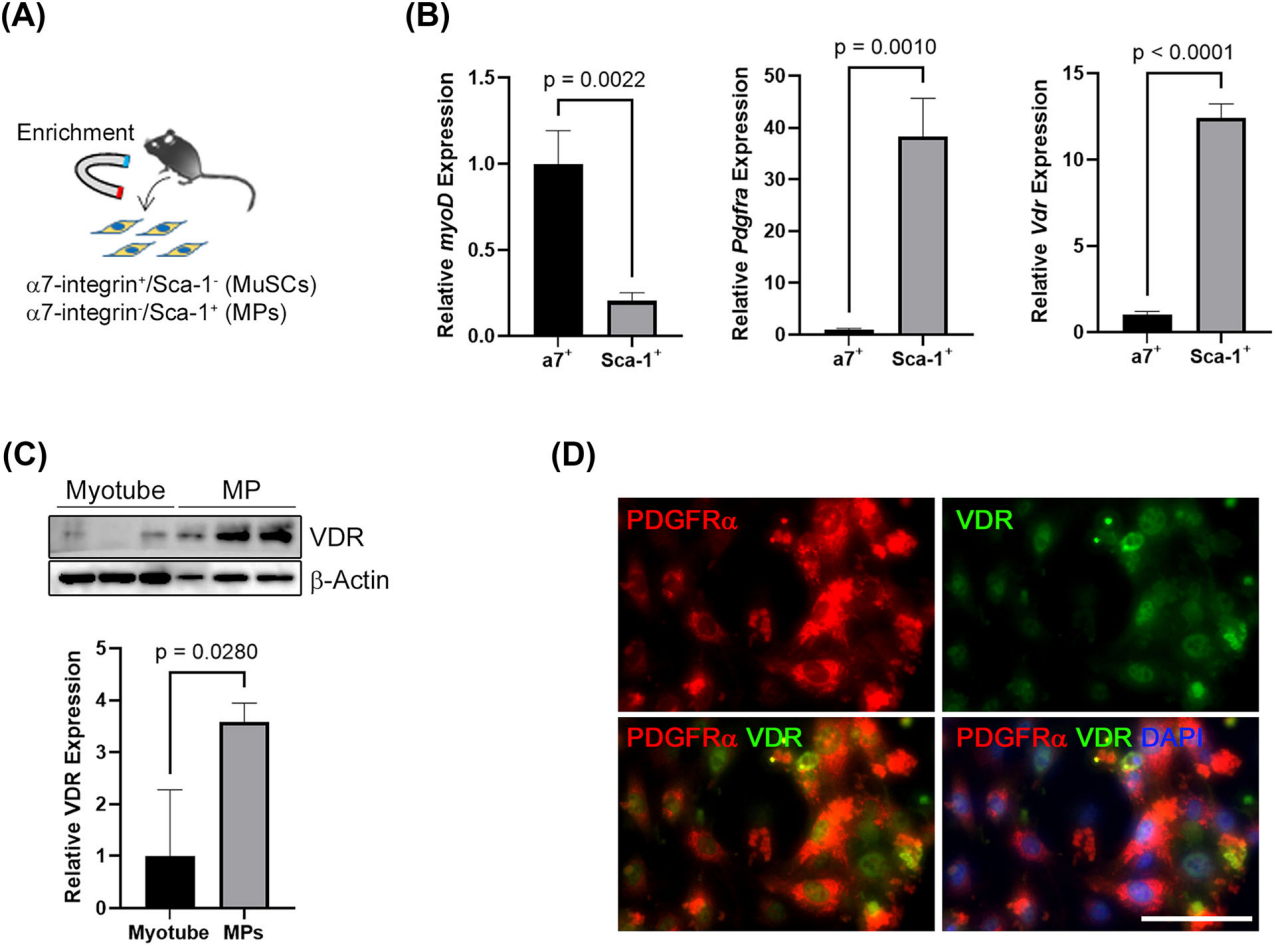
Mesenchymal progenitors (MPs) express the vitamin D receptor (VDR). (A) Scheme for the enrichment of mouse muscle stem cells and MPs using a magnetic separation system. Muscle stem cells and MPs were defined as the α7‐integrin^+^/Sca‐1^−^ and α7‐integrin^−^/Sca‐1^+^ fraction, respectively. (B, C) VDR mRNA and protein levels were significantly higher in MPs than in myogenic cells. (D) Immunocytochemistry for PDGFRα (red) and VDR (green) in primary mouse MPs. Scale bar = 100 μm.

To examine whether VDR is expressed in non‐myogenic cells in skeletal muscle, muscle stem cells and MPs were isolated from mouse skeletal muscles using a magnetic bead‐based cell isolation system (Figure 2A). Isolated mouse muscle stem cells and MPs were identified based on the expression of *myoD* and *Pdgfra* respectively, and VDR expression was compared between the two cell types. VDR mRNA and protein levels were significantly higher in MPs than in myogenic cells, including multinucleated myotubes (Figure 2B,C). Immunofluorescence analysis confirmed VDR expression in proliferating MPs (Figure 2D). These results suggested that MPs are newly identified vitamin D‐responder cells in adult skeletal muscle.

### Vitamin D inhibits adipogenesis of mesenchymal progenitors through direct regulation of early adipogenic factors

**Figure 3:**
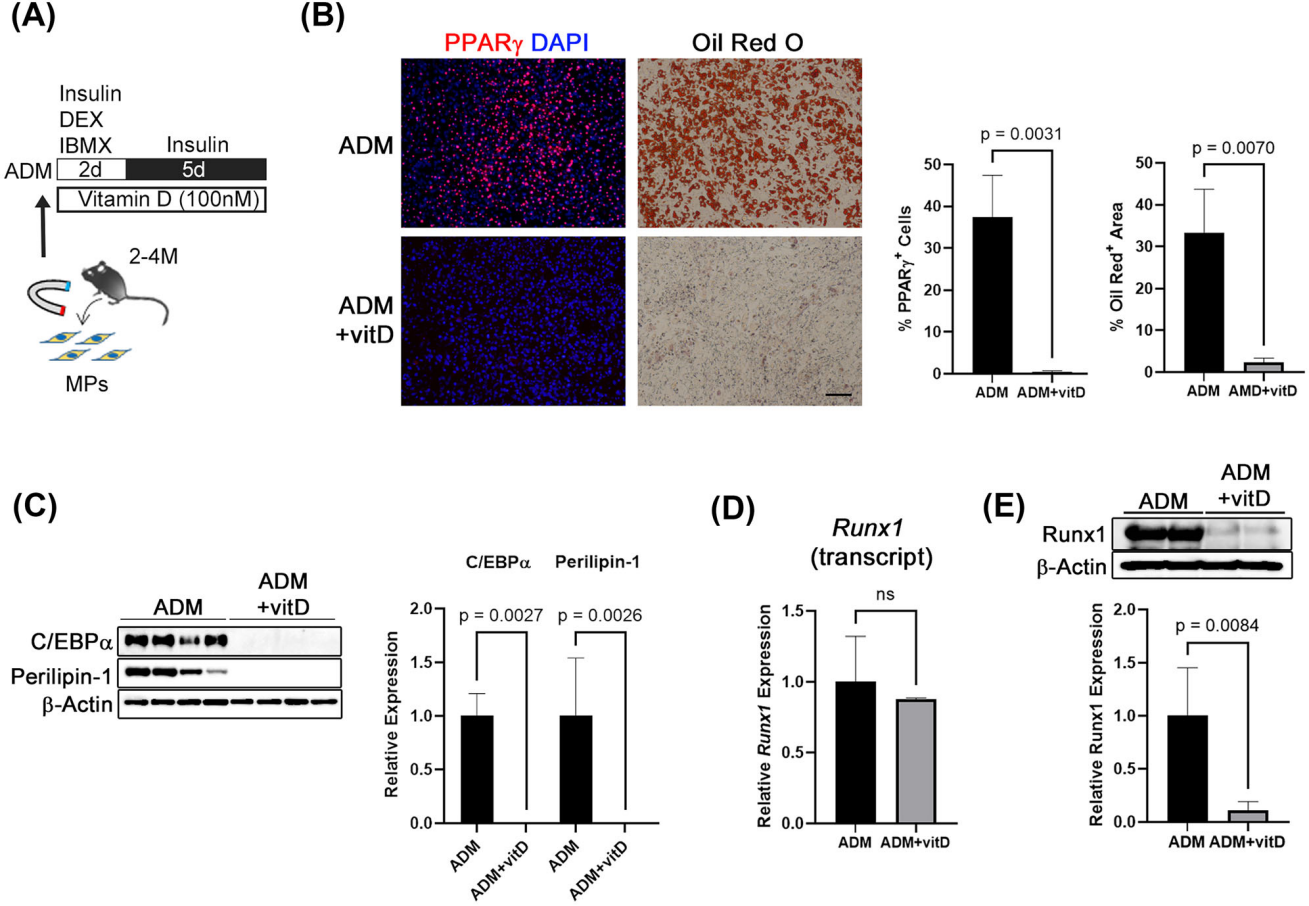
Vitamin D inhibits adipogenesis of MPs by obstructing Runx1‐mediated inhibition of early adipogenic factors. (A) α7‐integrin^−^/Sca‐1^+^ MPs were enriched from mouse hindlimb muscles (2–4 months of age) using a magnetic separation system. The mouse MPs were cultivated in medium containing insulin, dexamethasone, and 3‐isobutyl‐1‐methylxanthine for 2 days and then in medium containing insulin for 5 days in the presence or absence of vitamin D (100 nM). ADM, adipogenic differentiation medium. (B, C) Vitamin D treatment nearly completely inhibited adipogenesis of MPs. PPARγ^+^ and Oil red O‐stained adipocyte levels were significantly decreased in vitamin D‐treated MPs (ADM + vitD). Consistent herewith, the expression levels of C/EBPα and perilipin‐1 were significantly decreased in the ADM‐vitD group. *N* = 4. Scale bar = 100 μm. (D) Vitamin D treatment significantly decreased Runx1 protein in MPs, whereas gene expression was not affected. *N* = 4.

To clarify the action of vitamin D on MP dynamics, isolated mouse MPs were cultivated in growth medium containing vitamin D for 24 h. We observed no alterations in *mKi67* expression and EdU incorporation upon vitamin D treatment, suggesting that vitamin D did not affect the proliferation of MPs (Figure S1). Next, mouse MPs were cultivated in ADM with or without vitamin D for 7 days to investigate whether vitamin D would affect MP adipogenesis (Figure 3A). The numbers of PPARγ^+^ or Oil Red O^+^ MPs were significantly decreased upon vitamin D treatment, in line with C/EBPα and perilipin‐1 expression levels (Figure 3B,C), indicating that vitamin D exerts an inhibitory effect on MP adipogenesis. Further, vitamin D treatment decreased Runx1 protein expression, but not gene expression. As Runx1 is an upstream transcriptional regulator of early adipogenic factors such as PPARγ and C/EBPα,^11^ the inhibitory effect of vitamin D on adipogenesis was mediated by Runx1 protein regulation (Figure 3D). The inhibitory effect of vitamin D on MP adipogenesis was observed in aged mice‐derived MPs (Figure S2), suggesting that role of vitamin D signalling in MPs is universal, regardless of age.

### A vitamin D‐deficient diet causes fat accumulation in the muscles of aged mice, but not young mice

**Figure 4:**
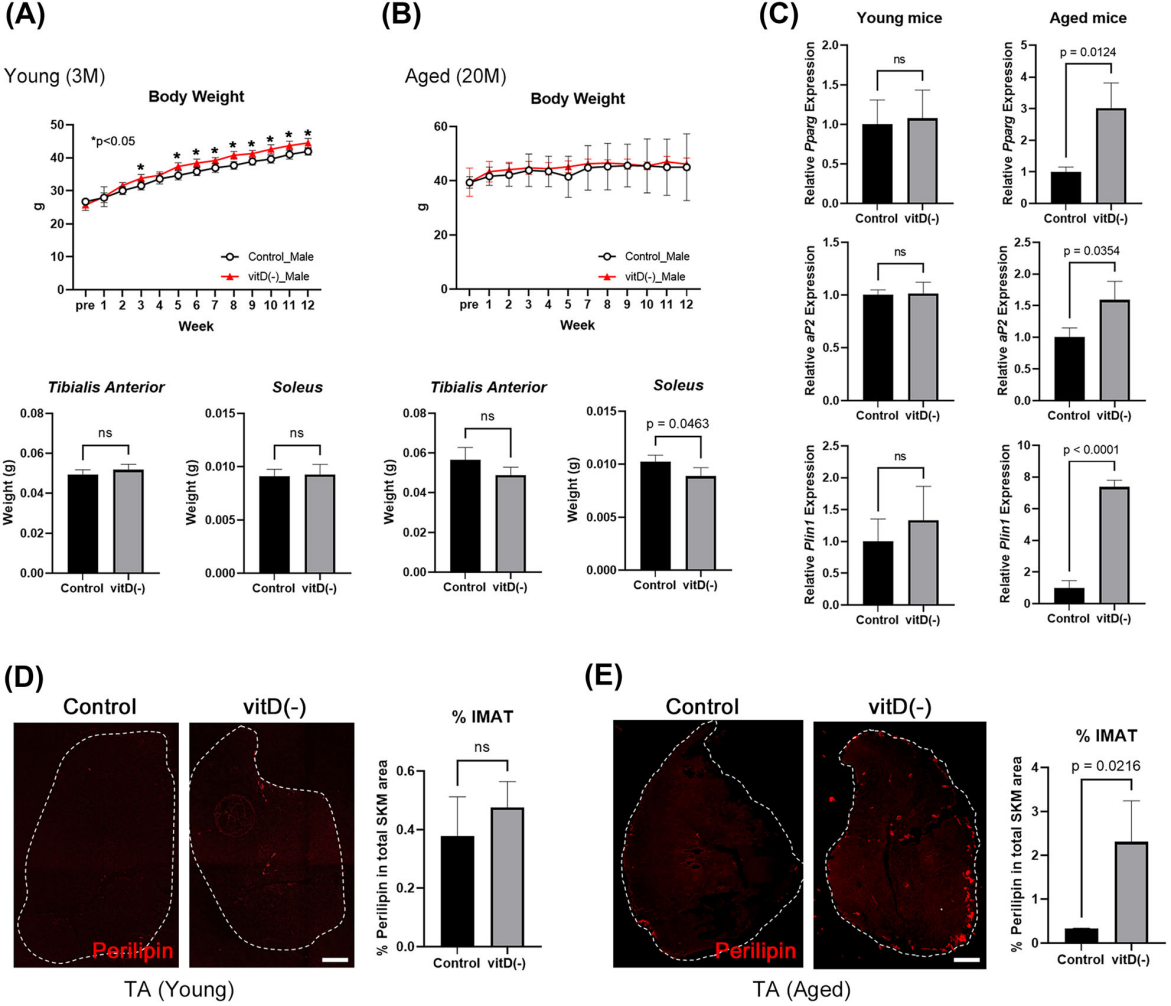
A vitamin D‐deficient diet induces IMAT in aged mice. (A, B) Young (3 months of age) and aged (20 months of age) male mice were given a vitamin D‐deficient (vitD^−^) or normal (control) diet for 3 months. Body weight was significantly increased in the young‐vitD^−^ group at some time points, whereas there was no significant difference in aged mice at any time point. Muscle weight at the end of the experiment was not significant different between control and vitD^−^ mice, except for soleus muscle weight, which was significantly decreased in aged mice. *P* < 0.05. *N* = 5 (at pre). (C) A vitamin D‐deficient diet increased expression of adipogenesis‐related genes in aged mice. *N* = 5. (D, E) A vitamin D‐deficient diet induced IMAT formation in aged mice (*P* = 0.0216, TA muscle), but not in young mice. IMAT was visualized by perilipin staining on muscle cross sections. *N* = 3. Scale bar = 500 μm.

Young (3 months of age) and aged (20 months of age) mice were fed a vitamin D‐deficient diet for 3 months, and body weight changes were monitored weekly. Body weight was significantly increased at several time points in the young mice on the vitamin D‐deficient diet, whereas no alterations were observed in the aged mice at any time point (Figure 4A,B). Skeletal muscles were dissected at the endpoint of this experiment (after 3 months of vitamin D‐deficient diet feeding) to evaluate tissue weight and fatty infiltration into the muscle. TA muscle weight was not affected in both young and aged mice, whereas soleus muscle weight was decreased in the aged mice on the vitamin D‐deficient diet (Figure 4A). Adipogenesis‐related genes such as *Pparg*, *aP2*, and *Plin1* were increased in expression in the aged mice on the vitamin D‐deficient diet (Figure 4C). In addition, intramuscular fat was observed only in aged mice on the vitamin D‐deficient diet (Figure 4D,E). These results suggested that vitamin D deficiency induces IMAT formation in the aged, but not the young muscle environment.

### The adipogenesis‐inhibitory effect of vitamin D is mediated by vitamin D receptor

**Figure 5:**
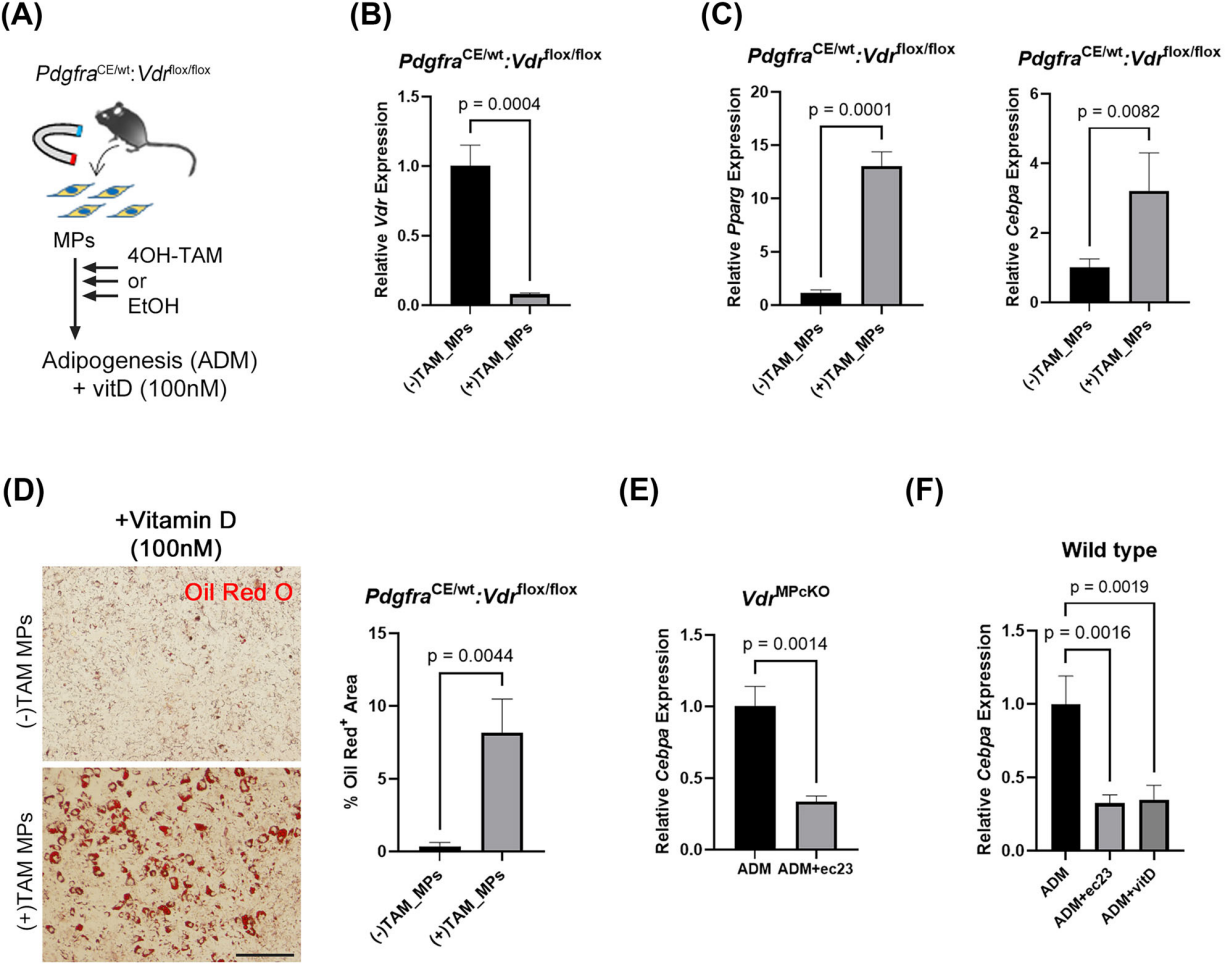
Defect of VDR in MPs suppresses the inhibitory effect of vitamin D on MP adipogenesis. (A, B) MPs were enriched from muscles of *Pdgfra* ^CE/wt^: *Vdr* ^flox/flox^ mice, and 4‐hydroxy‐tamoxifen was added to the medium for 3 days to defect VDR in MPs. *Vdr* expression in MPs was decreased upon tamoxifen treatment. (C) *Pparg* and *Cebpa* expression was increased in VDR‐deficient MPs. (D) Oil red O‐positive mature adipocyte levels were significantly increased in VDR‐deficient MPs. Scale bar = 100 μm. (E, F) synthetic retinoid (ec23) inhibited adipogenesis of MPs even under VDR deficiency. *N* = 3.

To investigate whether the inhibitory effect of vitamin D on MP adipogenesis depends on VDR, we developed tamoxifen‐inducible MP‐specific VDR‐deficient mice (*Pdgfra* ^CE/wt^: *Vdr* ^flox/flox^). MPs from this mouse line were treated with tamoxifen for 3 days to induce *Vdr* loss specifically in MPs and then cultivated in ADM with vitamin D for an additional 7 days (Figure 5A,B). The inhibitory effect of vitamin D on adipogenesis was cancelled in VDR‐deficient MPs, as adipogenesis‐related gene expression and Oil Red O^+^ adipocytes were significantly increased in the tamoxifen‐treated MP group (Figure 5C,D), indicating that vitamin D acts on MPs through a VDR‐mediated genomic pathway. A recent study demonstrated that retinoic acid, a vitamin A metabolite, inhibits adipogenesis of MPs in a manner similar to that of vitamin D.^22^ Therefore, we investigated whether vitamin D acts as an adipogenesis inhibitor independently from vitamin A. VDR‐deficient (*Vdr* ^MPcKO^) MPs were cultivated in ADM with photostable synthetic retinoic acid (ec23), and adipogenesis was evaluated based on the expression of adipogenic markers, such as *Cebpa*. Retinoic acid treatment inhibited MP adipogenesis even under VDR deficiency, and the inhibitory effect of retinoic acid was similar to that of vitamin D in wild‐type mouse‐derived MPs (Figure 5E,F). Therefore, vitamin D inhibits MP adipogenesis via a pathway independent from that of vitamin A.

### Mesenchymal progenitor specific vitamin D receptor deficiency induced IMAT formation in atrophied skeletal muscle

**Figure 6:**
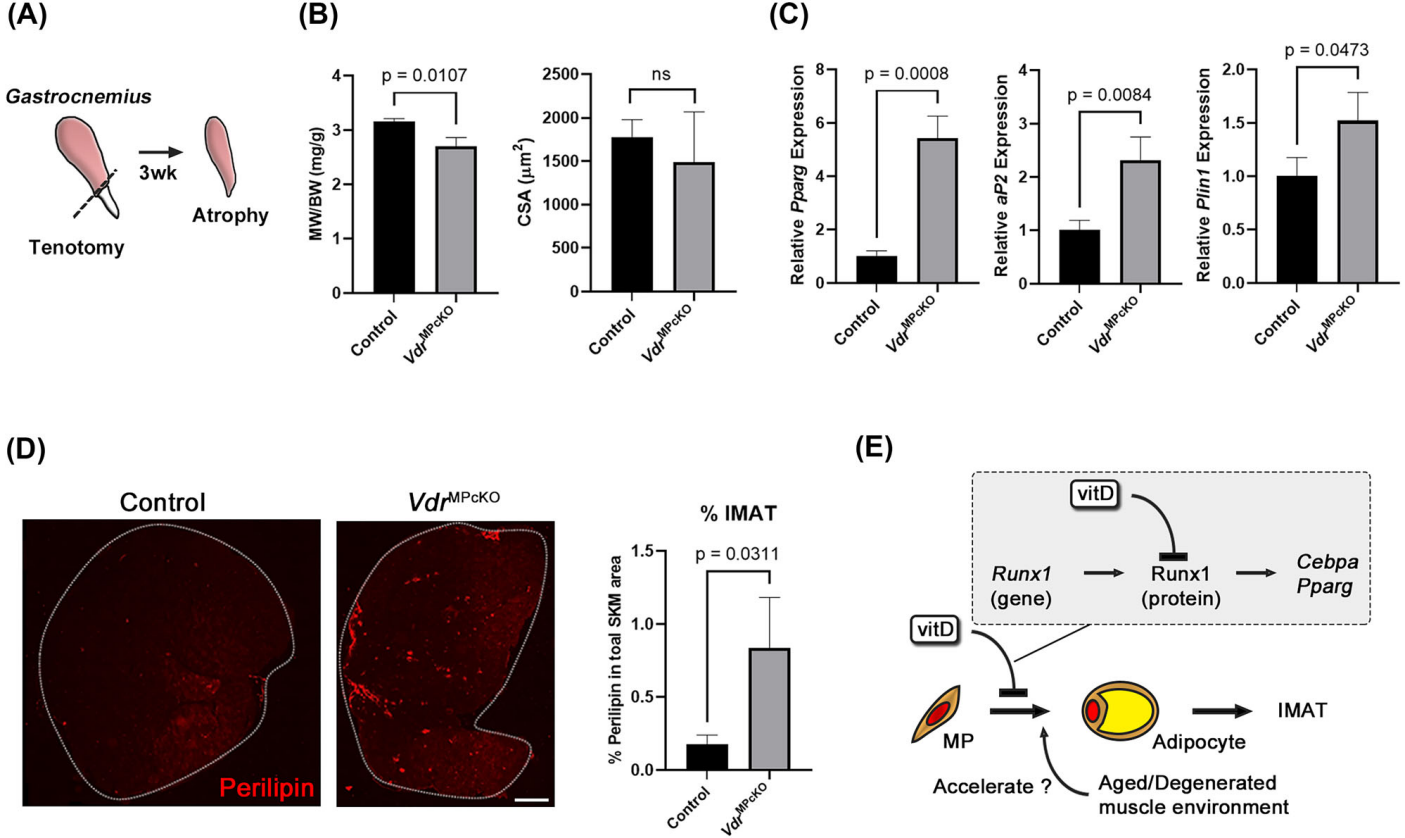
MP‐specific VDR deficiency causes IMAT formation in atrophied muscle. (A) Tenotomy model. Achilles tendon of gastrocnemius muscle was transected, and atrophied muscles at 3 weeks after the operation were used for experiments. (B) Muscle weight and cross‐sectional area of the gastrocnemius were compared between control and *Vdr* ^MPcKO^ mice. *N* = 4. (C) Expression levels of adipogenic markers including *Pparg*, *aP2*, and *Plin1* in atrophied muscles were significantly increased in *Vdr* ^MPcKO^ mice. *N* = 3. (D) *Vdr* ^MPcKO^ mice formed IMAT in atrophied muscle. The perilipin‐positive area in total muscle area was calculated. *N* = 3. Scale bar = 500 μm. (E) Suggested mechanism of the inhibitory effect of vitamin D on MP adipogenesis. Vitamin D inhibits the expression of early adipogenic genes by downregulating Runx1 protein. Aged or degenerated muscle may accelerate adipogenesis of MPs in vitamin D‐deficient conditions.

To induce muscle atrophy, *Vdr* ^MPcKO^ and tamoxifen non‐treated *Pdgfra* ^CE/wt^: *Vdr* ^flox/flox^ (control) mice were subjected to tenotomy of the gastrocnemius muscle (Figure 6A). At 3 weeks after the operation, muscle weight, myofibre cross‐sectional area, adipogenic marker gene expression, and fatty infiltration into the muscle were evaluated to clarify whether VDR deficiency in MPs induced IMAT formation *in vivo*. Tenotomy induced muscle weight loss in *Vdr* ^MPcKO^ mice, whereas the myofibre cross‐sectional area did not significantly differ between the two groups (Figure 6B). As tenotomy itself induces muscle atrophy,^20^ VDR deficiency in the MPs may have caused certain pathological changes in the muscle. In fact, Adipogenesis‐related gene expression was significantly increased in atrophied muscle of *Vdr* ^MPcKO^ mice, and the perilipin^+^ area in muscle cross sections was significantly higher in *Vdr* ^MPcKO^ mice than in control mice (Figure 6C,D). These findings indicated that MP‐specific VDR deficiency resulted in fatty infiltration into the muscle, in line with the findings in aged mice on a vitamin D‐deficient diet.

## Discussion

In most clinics, IMAT is evaluated using non‐invasive imaging techniques, such as magnetic resonance imaging, computed tomography, or dual‐energy X‐ray absorptiometry. Such evaluations have revealed that IMAT is frequently formed in older adults and gradually increases with age.^23^ However, these imaging tools are inconvenient to apply to thin muscles, such as the vastus medialis and vastus lateralis muscles, and it is difficult to accurately estimate age‐dependent changes in fat accumulation in these muscles. Although histological analysis for ectopic fat accumulation in vastus medialis muscle from OA patients has been performed using similar procedures,^17^ age‐related changes in fat infiltration was not estimated. In the present study, we histologically analysed fat accumulation in both the vastus medialis and vastus lateralis, with particular attention to the relationship with aging. We found that IMAT infiltration was positively correlated and significantly increased with age. These findings were consistent with those of conventional non‐invasive imaging evaluations of thick muscles, indicating the validity and accuracy of histological evaluations for IMAT. As OA itself causes muscle degeneration and fat accumulation in skeletal muscle,^17^ age‐related changes in the body may boost IMAT formation together with OA. It is widely known that the nutritional status changes with advancing age due to a substantial decline in food intake.^24^ Moreover, the levels of nutrients such as vitamin D, which is synthesized by the body in addition to dietary intake, decreased with advancing age.^25^ Given that vitamin D is associated with muscle physiology,^13^, ^26^ the decrease in vitamin D levels in older adults may be an additional factor contributing to IMAT formation in OA and other musculoskeletal diseases. In fact, this study revealed that vitamin D exerted a powerful inhibitory effect on the adipogenesis of MPs isolated from mouse skeletal muscle, indicating that the age‐related decrease in vitamin D production may be correlated with the increase in IMAT with advancing age. One limitation of this study is that we did not perform correlation analysis between blood vitamin D levels and IMAT infiltration in the study subjects because data on blood vitamin D levels were lacking. Therefore, further investigations focusing on the association between blood vitamin D levels and IMAT volume are needed.

VDR is expressed in various cells in the body, and VDR‐expressing cells are recognized as vitamin D‐responder cells. In skeletal muscle, myoblasts and myofibres are considered major vitamin D responders because they express VDR, and most studies on the role of vitamin D in skeletal muscle have used myogenic cells and myofibres.^12^, ^13^, ^27^ In fact, studies using myogenic cells have unveiled the actions of vitamin D in multiple steps of myogenesis, and it has been demonstrated that vitamin D contributes to muscle exertion by regulating contraction‐related factors in postnatal muscle.^13^ We newly discovered that VDR is expressed in MPs, clearly indicating that MPs are vitamin D‐responder cells in postnatal skeletal muscle and that vitamin D acts on not only myogenic cells but also non‐myogenic cells, such as MPs, in muscle. Interestingly, vitamin D signalling has quite different roles in MPs than in myogenic cells and plays an inhibitory role in adipogenic lineage commitment and subsequent IMAT formation. Although conventional studies on vitamin D mostly use myogenic cells, our findings offer another option for research on the actions of vitamin D in postnatal muscle. Given that vitamin D treatment inhibited adipogenesis even in aged mouse‐derived MPs, it can be expected that the inhibitory effect of vitamin D on MP adipogenesis is independent of age, and vitamin D supplementation is a therapeutic option to prevent IMAT formation in older adults.

As mentioned above, circulating vitamin D levels decrease with age due to an age‐related decline in vitamin D3 production in the skin.^28^, ^29^ This suggests that the suppressive effect of vitamin D on MP adipogenesis may be weakened in older adults, resulting in fat accumulation and expansion in the skeletal muscle. However, the mechanism underlying IMAT formation may be more complex as feeding a vitamin D‐deficient diet did not increase adipogenic expression and IMAT formation in young mice, whereas these were increased in aged mice, suggesting that vitamin D deficiency alone is not sufficient for IMAT formation *in vivo*. In addition, IMAT formation in *Vdr* ^MPcKO^ mice was observed only in atrophy‐induced muscle, not in contralateral normal muscle (data not shown). These results suggest that MP adipogenesis and subsequent IMAT formation are accelerated when vitamin D deficiency co‐occurs with specific muscle conditions such as atrophy and degeneration, as miscommunication with myofibres causes adipogenesis of intramuscular cells and myofibre degeneration is expected to continuously occur in older adults.^30^, ^31^ This is consistent at least in part with the findings of our pathological analysis using muscle cross sections of OA patients. Conversely, young or healthy muscle environments, characterized by well‐developed and non‐degenerated muscle, may prevent MP adipogenesis and IMAT formation even in vitamin D‐deficient circumstances.

The suppressive effect of vitamin D on MP adipogenesis is mediated by a VDR‐dependent genomic pathway, not a non‐genomic pathway, as *Vdr* ^MPcKO^ mouse‐derived MPs gave rise to adipogenic cells even in the presence of vitamin D. This suggests that the ligand‐bound VDR/RXR complex transcriptionally controls target gene expression to inhibit adipogenic differentiation of MPs. Initially, we assumed that vitamin D directly regulates the transcription of early adipogenic genes such as *Pparg* and *Cebpa* via the VDR‐dependent genomic pathway. In 3T3L1 preadipocytes, vitamin D directly inhibited the expression of both *Pparg* and *Cebpa*.^32^ However, genome‐wide analysis using human mononuclear cells did not identify the VDR‐binding site in these early adipogenic genes.^33^, ^34^ This suggested indirect transcriptional regulation of early adipogenic genes in the VDR‐dependent genomic pathway. However, our results demonstrated that vitamin D targeted Runx1, which is a transcription factor of adipogenic genes, including *Pparg* and *Cebpa*,^11^ and a decrease in Runx1 was a key event in the vitamin D‐mediated suppression of MP adipogenesis. Interestingly, we also found that vitamin D decreased Runx1 protein levels, but not transcript levels in adipogenesis‐induced MPs, suggesting that the target of vitamin D in MP adipogenesis is Runx1 translation or protein stability. As the latter are controlled by E3 ubiquitin ligases such as NEDD4 and CHIP/STUB1^35^, ^36^ and microRNAs such as miR‐206 in MPs,^11^ it is expected that vitamin D post‐transcriptionally regulates Runx1 to inhibit MP adipogenesis. In preliminary experiments, NEDD4 and CHIP/STUB1 expression were not significantly affected by vitamin D treatment in adipogenesis‐induced MPs (Figure S3A), suggesting that known Runx1‐associated ubiquitin ligases are not involved in the decrease in Runx1 protein levels in vitamin D‐treated MPs. However, we were not able to detect alterations in miR‐206 expression because of the undetectable levels of this microRNA in MPs in our preliminary experiment. Although further investigations are needed, the facts that Runx1 expression is regulated by several types of miRNAs and that vitamin D or VDR targets miRNAs^37^, ^38^ suggest post‐transcriptional regulation of Runx1 by vitamin D‐mediated miRNAs in MPs.

Recent studies have demonstrated that the administration of retinoic acid or a retinoic acid receptor agonist suppressed MP adipogenesis and subsequent IMAT formation in mice,^22^, ^39^ indicating that vitamin A and retinoid signalling play an inhibitory role in adipogenic differentiation of MPs, similar to vitamin D. Indeed, using the synthetic retinoic acid agonist ec23, we confirmed an inhibitory effect of retinoic acid similar to that of vitamin D on MP adipogenesis. As the inhibitory effect of retinoic acid was demonstrated even in VDR‐deficient MPs, vitamin A and vitamin D are expected to act on MP adipogenesis and IMAT formation via independent inhibitory pathways. While increased expression of *Pref‐1*, *Sox9*, and *Klf2*, which encode molecular targets of the retinoic acid/retinoic acid receptor complex in MPs, is important to inhibit MP adipogenesis,^22^ vitamin D did not increase the expression of these factors, except for *Pref‐1*, in adipogenic conditions (Figure S3B). This result supports the idea that vitamin A and D suppress MP adipogenesis via different molecular pathways. In brief, vitamin D suppresses MP adipogenesis via Runx1‐mediated inhibition of early adipogenic genes and upregulation of Pref‐1, which is constitutively expressed in undifferentiated MPs.^7^

In summary, our study revealed that vitamin D plays an inhibitory role in the adipogenic differentiation of MPs and that lack of vitamin D signalling may promote MPs to give rise to adipocytes and subsequently form IMAT in adult skeletal muscle. Importantly, IMAT formation may be prevented in ‘healthy’ skeletal muscle even under vitamin D deficiency or impaired VDR signalling, indicating the existence of an additional factor(s) contributing to IMAT formation in addition to vitamin D deficiency. Given that IMAT was expanded in aged and atrophied skeletal muscle in the present study, aging‐related muscle changes such as atrophy and degeneration are candidate factors contributing to ectopic fat formation in muscle. Similarly, fat tissue in muscles of OA patients, who generally have severe muscle degeneration due to long‐term OA pathology,^17^ increases with age. Further, our study revealed that the inhibitory effect of vitamin D on MP adipogenesis is mediated by Runx1, a transcription factor of early adipogenic genes. However, how exactly vitamin D regulates Runx1 protein expression in MPs remains to be investigated. Our findings further elucidate the role of vitamin D in muscle homeostasis and suggest that vitamin D supplementation may be a therapeutic option to prevent IMAT and sarcopenia in older adults.

## Conflict of interest

Dr. Tohru Hosoyama received funding from Zenyaku Kogyo, Co., Ltd. The other authors declare no conflicts of interest.

## Supporting information


**Figure S1.** Vitamin D does not affect MP proliferation. (A) Primary mouse MPs were cultivated in growth medium with vitamin D and EdU for 24 h. The effect of vitamin D treatment on proliferation was evaluated based on mKi67 expression and EdU incorporation. (B) Vitamin D did not affect MP kinetics. There were no statistically significant differences in mKi67 expression in MPs and the number of EdU + proliferating MPs. ns: no statistical significance.
**Figure S2.** Vitamin D inhibits adipogenesis of aged mouse‐derived MPs. (A) Expression levels of adipogenic genes were significantly decreased in vitamin D‐treated MPs derived from 30‐month‐old mice. *N* = 3. (B) Both PPARγ+ and Oil‐Red O + adipocytes were significantly decreased in vitamin D‐treated MPs derived from 30‐ month‐old mice. N = 3. Scale bar = 100 μm.
**Figure S3.** Expression of Runx1‐associating factors in MPs in the presence or absence of vitamin D. (A) Runx1 degradation‐associated E3 ubiquitin ligases, NEDD4 and CHIP/STUB1, in MPs are not affected by vitamin D treatment. (B) Vitamin D does not increase RA/RXR‐associated adipogenesis‐inhibitory factors, Sox9 and Klf2, while Pref‐1 was significantly increased in vitamin D‐treated MPs. *N* = 3.
