# Supplementary material for: Lack of vitamin D signalling in mesenchymal progenitors causes fatty infiltration in muscle
Source: J Cachexia Sarcopenia Muscle. 2024 Mar 27;15(3):907–18. doi: 10.1002/jcsm.13448 (PMC11154772; doi:10.1002/jcsm.13448)
Supplement: Supplementary file 1 — Figure S1. Vitamin D does not affect MP proliferation. (A) Primary mouse MPs were cultivated in growth medium with vitamin D and EdU for 24 h. The effect of vitamin D treatment on proliferation was evaluated based on mKi67 expression and EdU incorporation. (B) Vitamin D did not affect MP kinetics. There were no statistically significant differences in mKi67 expression in MPs and the number of EdU + proliferating MPs. ns: no statistical significance. Figure S2. Vitamin D inhibits adipogenesis of aged mouse‐derived MPs. (A) Expression levels of adipogenic genes were significantly decreased in vitamin D‐treated MPs derived from 30‐month‐old mice. N = 3. (B) Both PPARγ+ and Oil‐Red O + adipocytes were significantly decreased in vitamin D‐treated MPs derived from 30‐ month‐old mice. N = 3. Scale bar = 100 μm. Figure S3. Expression of Runx1‐associating factors in MPs in the presence or absence of vitamin D. (A) Runx1 degradation‐associated E3 ubiquitin ligases, NEDD4 and CHIP/STUB1, in MPs are not affected by vitamin D treatment. (B) Vitamin D does not increase RA/RXR‐associated adipogenesis‐inhibitory factors, Sox9 and Klf2, while Pref‐1 was significantly increased in vitamin D‐treated MPs. N = 3. [file JCSM-15-907-s001.pdf]

Supplemental Figure 1.

A

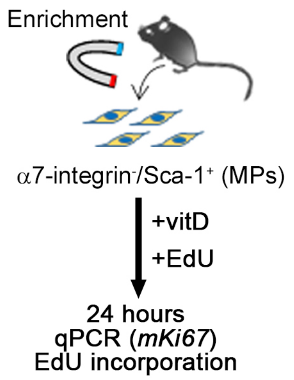

B

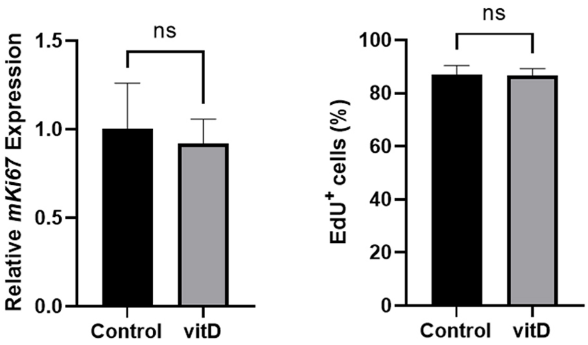

Supplemental Figure 2.

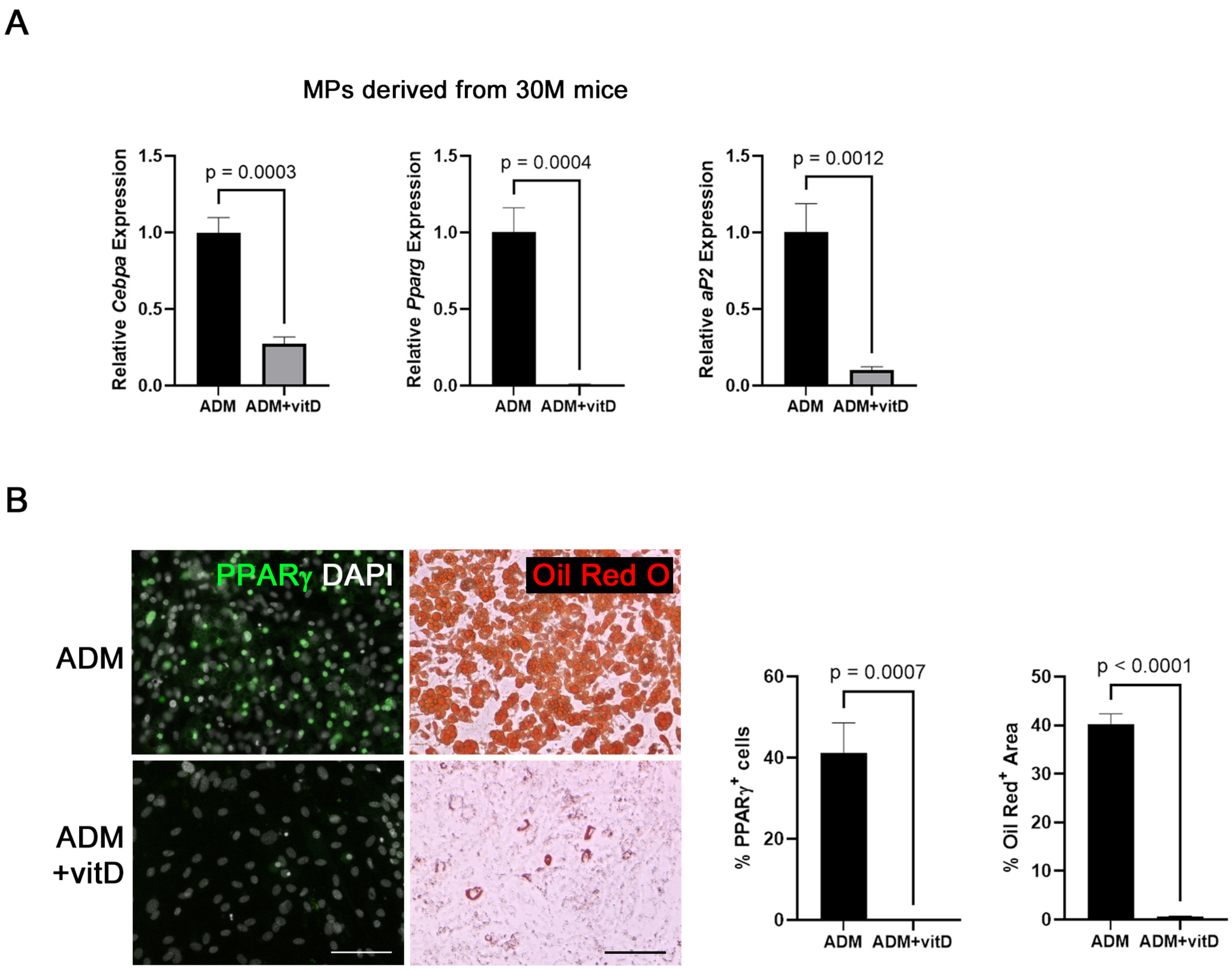

Supplemental Figure 3.

A

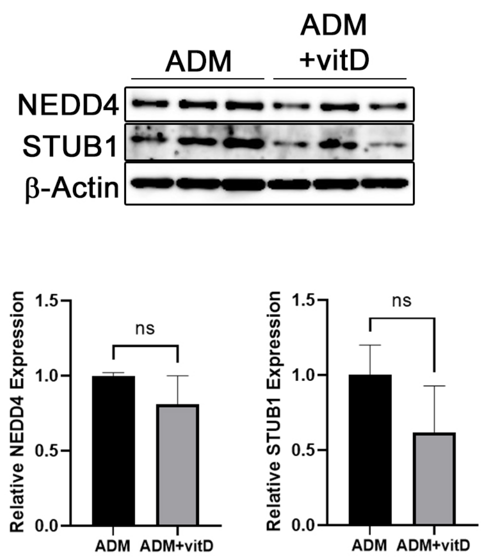

B

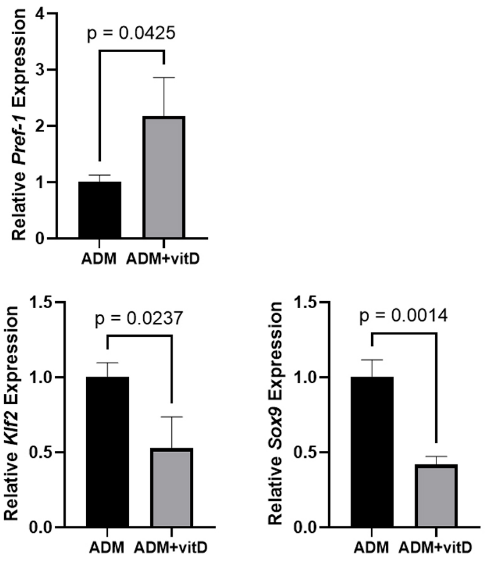

Supplemental Figure 1. Vitamin D does not affect MP proliferation. (A) Primary mouse MPs were cultivated in growth medium with vitamin D and EdU for 24 h. The effect of vitamin D treatment on proliferation was evaluated based on *mKi67* expression and EdU incorporation. (B) Vitamin D did not affect MP kinetics. There were no statistically significant differences in *mKi67* expression in MPs and the number of EdU^+ proliferating MPs. ns: no statistical significance.

Supplemental Figure 2. Vitamin D inhibits adipogenesis of aged mouse-derived MPs. (A) Expression levels of adipogenic genes were significantly decreased in vitamin D-treated MPs derived from 30-month-old mice. N = 3. (B) Both $PPAR\gamma^+$ and Oil-Red O^+ adipocytes were significantly decreased in vitamin D-treated MPs derived from 30-month-old mice. N = 3. Scale bar = 100 μm.

Supplemental Figure 3. Expression of Runx1-associating factors in MPs in the presence or absence of vitamin D. (A) Runx1 degradation-associated E3 ubiquitin ligases, NEDD4 and CHIP/STUB1, in MPs are not affected by vitamin D treatment. (B) Vitamin D does not increase RA/RXR-associated adipogenesis-inhibitory factors, *Sox9* and *Klf2*, while *Pref-1* was significantly increased in vitamin D-treated MPs. N = 3.
